# Side Streams of Broccoli Leaves: A Climate Smart and Healthy Food Ingredient

**DOI:** 10.3390/ijerph17072406

**Published:** 2020-04-01

**Authors:** Emilia Berndtsson, Roger Andersson, Eva Johansson, Marie E. Olsson

**Affiliations:** 1 Department of Plant breeding, Swedish University of Agricultural Sciences, SE-230 53 Alnarp, Sweden; eva.johansson@slu.se; 2 Department of Molecular Sciences, Swedish University of Agricultural Sciences, SE- 750 07 Uppsala, Sweden; roger.andersson@slu.se

**Keywords:** broccoli, dietary fiber, gut microbiota, health, leaves, phenolic compounds, side steams

## Abstract

Human consumption of fruits and vegetables are generally below recommended levels. Waste from the production, e.g., of un-used parts such as broccoli leaves and stem when producing broccoli florets for food, is a sustainability issue. In this study, broccoli leaves were analyzed for the content of various dietary fibre and phenolics, applying the Uppsala method and HPLC analyses, respectively. The results showed that broccoli leaves had comparable levels of dietary fibre (26%–32% of dry weight (DW)) and phenolic compounds (6.3–15.2 mg/g DW) to many other food and vegetables considered valuable in the human diet from a health perspective. A significant positive correlation was found among soluble dietary fibre and phenolic acids indicating possible bindings between these components. Seasonal variations affected mainly the content of conjugated phenolics, and the content of insoluble dietary fibre. This study verified the importance of the use of broccoli production side streams (leaves) as they may contribute with health promoting components to the human diet and also socio-economic and environmental benefits to the bioeconomic development in the society.

## 1. Introduction

Human health benefits from diets being rich in fruits and vegetables have been verified in a range of studies, and is partly due to an association with a reduction in cardiovascular disease and cancer mortality [1]. Both fruit and vegetables, as well as other plant based foods, are rich in compounds that are suggested to have health beneficial properties [2]. Of these compounds, in particular dietary fiber and bioactive compounds such as phenolics are reported as beneficial when sufficiently consumed [3,4,5]. 

Dietary fiber is a term used for naturally occurring carbohydrate polymers that are not digested nor absorbed in the small intestine, and that have health beneficial properties [6]. Dietary fiber can be divided into two fractions, soluble (SDF) and insoluble (IDF) dietary fiber, due to the solubility in water. Most plant foods contain a combination of SDF and IDF [7]. Dietary fiber has been shown to promote health benefits, such as lowering cholesterol in the blood [8], have an impact on the rate of gastric emptying [9], and promote peristaltic movement in the intestines [10]. In addition, dietary fiber is important as energy source for the gut microbiota, which will use the dietary fiber to produce short chained fatty acids (SCFA) [11]. These SCFA can be absorbed and can help in regulating the metabolism and immune system of the host [11]. A diet that contains several types of dietary fiber has been suggested to lead to a gut microbiota with an increased diversity, which in turn could have health beneficial effects [11,12,13]. Fruit and vegetables have been shown to be good sources of dietary fiber [14]. The edible parts of vegetables in the Brassica family usually contain dietary fiber in moderate to high amounts [15,16,17]. Given the recent interest in increasing the resource efficiency by using side streams of different produce, broccoli leaves could be an attractive new source of dietary fiber. 

In earlier studies, dietary fiber and phenolic compounds have been analyzed separately due to substantial differences in their chemical structure and biological properties, even though the phenolic compounds that are associated with the dietary fiber might have a significant contribution to the overall health [18,19]. Dietary fiber is proposed to bind phenolics [20,21,22], enabling these compounds to escape digestive enzymes in the upper gastrointestinal tract and instead reach the colon intact [23,24]. There, the gut microbiota can ferment both the dietary fiber and the phenolics to more easily absorbable compounds. 

Phenolic compounds are defined as substances possessing an aromatic ring bearing one or more hydroxyl group including their functional derivatives [25]. In plants, the phenolic compounds have various functions, such acting as anti-feedant, anti-pathogenic, and protective agents (e.g., for UV light) [25]. They also provide pigmentation of plants, are attractants for pollinators, make the cell walls impermeable for gas and water, and contribute to physical stability of the plant [25]. Phenolic compounds are often complex molecules, that are transformed into molecules of smaller size by the gut microbiota before absorption, which increases the bioavailability of these compounds [26]. Most phenolic compounds have antioxidative properties, hence protecting the cells from, e.g., free radicals [27]. Furthermore, the phenolic compounds have been implicated as involved in improving the vascular health [28], lower the risk for developing certain types of cancer [29] and lower the risk of chronic inflammations [3,30]. Phenolic compound may also have an impact on the diversity of the gut microbiota, if they can reach the colon intact [26]. Leafy green vegetables usually contain high levels of phenolic compounds [31]. In Brassica vegetables, including broccoli, a large number of phenolic compounds have been identified [32,33,34], mainly from the parts already used as food, such as the broccoli florets and kale leaves. This indicates that broccoli leaves should contain phenolic compounds in comparable amounts. 

The florets in broccoli (*Brassica oleracea* Italica group) have been shown to contain health beneficial compounds, such as vitamin K and C, minerals, dietary fiber, phenolic compounds, glucosinolates and folic acid [35,36,37]. The broccoli leaves, on the other hand, are not as well studied as the florets, but have been shown to have higher levels of phenolic compounds as compared to the florets [38,39]. The stem in broccoli contains large amount of insoluble fiber and low amounts of soluble fiber [40]. 

From the currently applied greenhouse production systems of broccoli, it has been estimated that only 10% of the above ground biomass ends up as broccoli florets for consumption. The rest (90%) of the above ground broccoli plants (which includes stems, leaves, and inflorescences of insufficient size) becomes waste [41]. Previous experiments have shown that 70% of the total weight of the broccoli plants is wasted in the field, while 45–50% of the harvested edible broccoli florets are wasted during processing and transportation [42]. Such parts of the broccoli plant, today cultivated and edible but not used as food, are interesting sources for use as novel food products. These side streams have a potential to be used as functional ingredients to improve the nutritional values of different food products.

The aim of this study was to evaluate the content and composition of dietary fiber and phenolic compounds in broccoli leaves, and to investigate potential relationships between the content and composition of these groups of compounds. A second aim was to discuss possible impact on health from consumption of broccoli leaves, based on the evaluated content and composition of these compounds. Furthermore, the study aimed to describe possible food applications of broccoli leaves as a side stream from commercial broccoli (florets) production.

## 2. Materials and Methods 

### 2.1. Plant Material

Broccoli leaves were collected on the fields at a commercial production site located in the southern part of Sweden, in the vicinity of 56°24′38.5″N 12°39'34.5"E. The grower used the same broccoli cultivar ´Beneforte´, known for its nutritional high value [43], throughout the whole production site. The broccoli florets to be commercialized were harvested in October during the two years of sampling, 2017 and 2018. The leaves for this study were collected within 24 h after the final harvest of the broccoli florets. Leaves were collected from a total of four fields; two fields in 2017 and two fields in 2018 (denominated Field 1 (2017), Field 2 (2017), Field 3 (2018) and Field 4 (2018)). In each field, three squares (1.5 × 1.5 m) were randomly positioned (excluding edges of the fields) and ten plants were selected from each square. The plants were cut approximately 2 cm above ground, excluding the roots and most woody lower section. The plants were then transported to the lab in plastic bags, washed under flowing water to rinse away visible dirt, air dried and the whole leaves (including midvein and petiole) were thereafter placed pairwise in bags and stored at –80 °C to minimize the degradation of phenolic compounds.

### 2.2. Water Content Determination and Milling

Water content for analysis was determined by weighing the frozen samples before and after freeze-drying for 48 h. The freeze-dried samples were milled using an Ultra Centrifugal Mill ZM 200 (Retsch GmbH, Haan, Germany) equipped with a sieve with pore size <0.5 mm. The powder was stored in +4°C in dark plastic containers until analysis.

### 2.3. Analysis of Dietary Fibere

The components of dietary fiber were analyzed according to the Uppsala method [44], with modification according to Andersson et al. [45] for separate analysis of soluble and insoluble dietary fiber components (sugar residues); Klason lignin, uronic acid (UA), rhamnose (rha), fucose (fuc), arabinose (ara), xylose (xyl), mannose (man), galactose (gal) and glucose (glc). Following previous experiences and method descriptions [44,45], analysis were performed in duplicates. The analytical results are reported on a dry matter basis (DW). Dry matter was determined by drying the milled samples at 105 °C for 16 h. 

### 2.4. Analysis of Phenolic Compounds 

All samples were analyzed in triplicates, and measurements of phenolic compounds were according to Lin et al. [46], with some modifications as described below. Similar as in our previous study [47], a methanol extraction was applied as described below, following common practice for phenolic compounds [48,49,50].

#### 2.4.1. Methanol Extraction

For each sample, 2 mL 60% MeOH were added to 100 mg freeze-dried leaf sample in an Eppendorf tube and vortexed (Combi-spin FVL-2400, Biosan, Latvia) for 5 seconds until mixed. The tubes were put in ultrasonic bath (Sonorex Digitec DT 100 H, Bandelin, Germany) at 35 °C, for 60 min in order to extract the phenolic compounds from the tissues, and thereafter chilled shortly in cold water. The tubes were centrifuged at 4 °C and 21,000× *g* in a Centrifuge 5427 R (Eppendorf, Hamburg, Germany), for 10 min to separate sufficient supernatant from pellet. An aliquot of the supernatant was saved as methanol extract for analysis with HPLC, while one other aliquot was analysed further with alkaline hydrolysis. Compounds analysed from the methanol extraction are denominated as *conjugated phenolics*, since the phenolic compounds in Brassica are commonly found as conjugated to sugars and organic acids [51]. The conjugated phenolics normally include naturally occurring flavonoid glycosides and phenolic acids glycosides [51]. The conjugated phenolics were therefore further subdivided into two groups (called *Flavonoids* and *Phenolic Acids Derivatives*, respectively), based on their retention time in the chromatogram (Figure S1).

#### 2.4.2. Hydrolysis

After the methanol extraction, alkaline hydrolysis was used on the supernatant from samples in order to liberate the phenolic acids from their glycoside. 

For the alkaline hydrolysis, 200 µL 2 M NaOH was added to 500 µL supernatant from the methanol extraction for each sample and the tube was shortly vortexed to mix. Then, the tube was put on a shaking bed at 2 °C for hydrolysis during 18 h. Thereafter, 280 µL 6 M HCI was added and the tube was again vortexed. A liquid-liquid extraction was performed by adding 2 × 500 µL ethylacetate to extract the released phenolic compounds. The top phase was collected and the ethylacetate was evaporated under N_2_ until dryness. The residue was dissolved in 100 µL 100% MeOH and the tube was placed in ultrasonic bath, at 25 °C, for 5 min to dissolve the sample. An amount of 100 µL of the solution was transferred to a HPLC vial for analysis with HPLC. Compounds analyzed from the alkaline hydrolysis are denominated *phenolic acids*. The phenolic acids (after hydrolysis) were further subdivided into two groups (called Group 1 and Group 2) based on their retention time in the chromatogram (Figure S1).

#### 2.4.3. HPLC Analysis

In order to identify the phenolic compounds, the samples were analyzed by HPLC-MS. The phenolic compounds were identified by their particular spectra, their UV-maxima, molecular weight and retention time, and were compared with previous literature [32,33,34]. For the methanol extract, kaempferol-3-O-rutinoside (Extrasynthèse, France) was used as an external standard and for alkaline hydrolysis caffeic acid (Sigma, Germany) was used. 

The individual phenolic compounds were analyzed in a HPLC–DAD–ESI(-)–MS system, Agilent 1260 (Agilent Technologies, Waldbronn, Germany). The system consisted of binary pump (0.700 mL/min), thermostated column compartment (35 °C), with a Triart C_18_ ExRS column (YMS, 150 mm × 3 mm, particle size 3µm and pore size 8 nm), an autosampler, a diode array detector (DAD) (350 nm for methanol extract and 280 nm for alkaline hydrolysis), a mass spectrometer (Agilent 6120, ionization mode API-ES negative polarity, gas temperature of 350 °C, drying gas 12.0 L/min, m/z 130–800). Data acquisition was made with Chemstation software (B04.03-SP1 [version 87], Agilent Technologies, Waldbronn, Germany). Injection volume was 3.00 µL per sample. The mobile phase consisted of a binary solvent system using water acidified with 0.5% formic acid (solvent A) and 0.1% formic acid in acetonitrile (solvent B). The gradient increased linearly from 0–3% B (v/v) at 0–7 min, to 3–12% B at 7–13 min, to 12–14% B at 13–17 min, to 14–35% B at 17–26 min, held at 35% B at 26–28 min, decreased to 3% B at 28–32.5 min and held at 3% B at 32.5–35 min.

### 2.5. Statistical Analysis

Data were expressed as mean ± standard deviation (SD). Statistical analysis was made in RStudio Team (2016, US), version 1.1.456 [52], with the packages ggplot2, ggbiplot, dplyr, emmeans, lme4 and lmerTest.

Variation in content of different compounds in plants is related to variation among genotypes and environment of cultivation, know to play an equal role and being related to selection of cultivars and environments used [53,54]. It is known from a broad range of studies that environmental variation is due to multitude of factors including year, site and field variation, originating from variation in soil, temperature, precipitation etc. [55,56]. Comparisons of environmental effects on compounds evaluated in the study were carried out applying a general linear model analysis of variance (ANOVA) comparing effects of years and fields. When significant differences (*p* < 0.05) were found, the differences between the means were evaluated by the use of Tukey post-hoc test (build in the command compact letter display (CLD)). A principal component analysis (PCA) was made to investigate the relationship between content of dietary fiber and phenolic compounds. Each data point was the average from two (dietary fiber) or three (phenolic compounds) sample replications. Content of dietary fiber and phenolic compounds in the analyzed broccoli leaves were compared with content in other comparable food items with data collected from literature. Due to different levels of digits presented in various publications concerning this data, all numbers were rounded to one decimal level. 

## 3. Results

### 3.1. Dietary Fiber and Water Content in Broccoli Leaves

The majority of the dietary fiber in broccoli leaves consisted of IDF, comprising 23.8%–30.6% of the DW as compared to the SDF constituting 2% of the DW (Table 1). Cultivation location impacted the concentration of IDF in the leaves (23.8–30.6% of DW), and significant differences were found between Field 1 and 4 (Table 1). No significant differences were found for SDF and total dietary fiber (TDF) among fields, and neither among years for the total content of IDF, SDF, or TDF in the leaves (data not shown).

Significant differences were found for the content of dietary fiber constituents (Klason lignin and sugar residues) in samples originating from different fields and years. Similarly, as for the total content of dietary fiber, the content of the individual soluble fiber constituents was generally low in comparison with the content of insoluble fiber constituents. Among the analyzed dietary fiber (Table 2), the most abundant constituents were Insol glc, Insol UA, and Insol xyl. Significant differences were found among samples from different fields in content of individual constituents for Insol UA, Insol ara, Sol ara, Sol xyl, Sol man, and Sol glc (Table 2).

Higher content was found for leaves from 2017 as compared to those from 2018 of the insoluble fiber constituents Insol UA, Insol ara, and Insol man (Table 2). The content was instead lower in leaves from 2017 as compared to those from 2018 of some soluble fiber constituents Sol fuc, Sol xyl, Sol man, and Sol glc (Table 2). 

The water content in broccoli leaves, measured before and after freeze-drying of the samples, was approximately 80%, with 84.8 ± 1.5% in 2017, and 80.9 ± 2.9% in 2018.

### 3.2. Phenolic Compounds in Broccoli Leaves 

Year of cultivation impacted significantly the amount and composition of phenolic compounds in broccoli leaves. Thus, a significantly higher content of conjugated phenolics (compounds analyzed in methanol extract, mainly phenolic compounds conjugated to sugars and phenolic acids [51]) was found in leaves harvested in 2017 (10.8–15.2 mg/g DW) as compared to 2018 (6.3–7.5 mg/g DW), while the content of phenolic acids (compounds analyzed in methanol extract after alkaline hydrolysis) did not differ significantly in leaves harvested in different years (Table 3).

Thereby, similar amounts of conjugated phenolics and phenolic acids were found in leaves harvested in 2018, while 2.5-5 times higher levels of conjugated phenolics as compared to phenolic acids (after hydrolysis) were noted in leaves harvested in 2017. In addition, a second group of compounds was detected in the phenolic acids chromatogram in leaves from 2018, which were not found in those from 2017 (Figure S1). No significant difference was found neither in the content of conjugated phenolics, nor in content of phenolic acids in the broccoli leaves from the different fields.

### 3.3. Relationship among Dietary Fiber and Phenolic Compounds in Broccoli Leaves

**Figure 1:**
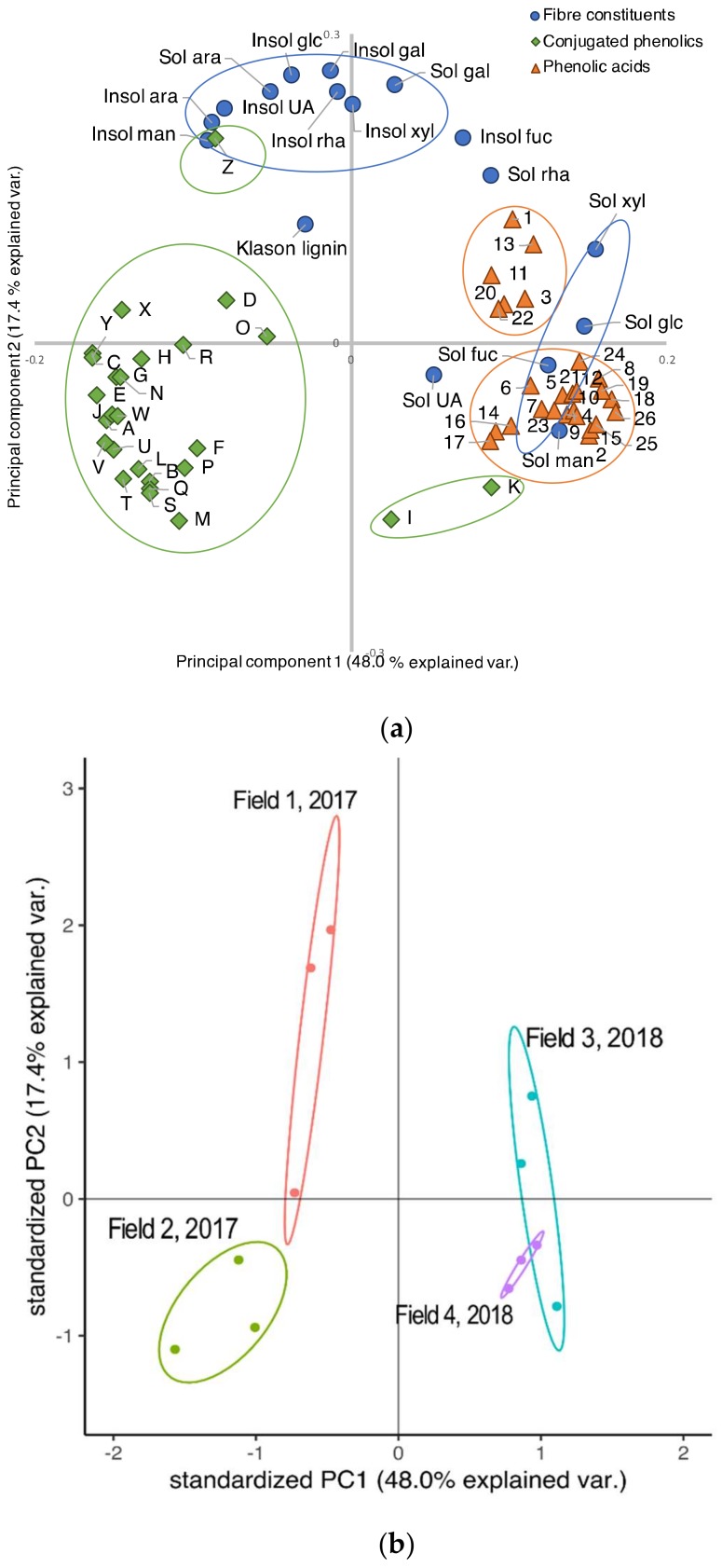
Loading plot (a) and score plot (b) for the principal component analysis for dietary fiber constituents (Klason lignin and sugar residues), conjugated phenolics and phenolic acids (after hydrolysis) from broccoli leaves. Each data point is the mean from three replications, n = 3. Insol: insoluble. Sol: soluble. UA: uronic acid. Rha: rhamnose. Fuc: fucose. Ara: arabinose. Xyl: Xylose. Man: mannose. Gal: galactose. Glc: glucose. For a tentative identification of the peaks in conjugated phenolics see Table 5. For the phenolic acids, Peaks 2, 4, 5, 8, 9, 10, 16 and 19 have a tentative identification (Table S1), and HPLC and MS spectra can be found in Figure S7 and Figure S8.

Principal component analysis visualized a close relationship among some of the soluble dietary fiber (Sol fuc, Sol xyl, Sol man, Sol glc) and the phenolic acids and also two of the conjugated phenolics (I and K), as could be seen from their positive values on PC1 from the loading plot (Figure 1b). In addition, a significant and positive Pearson correlation (*p* < 0.05) was found for the Group 1 of phenolic acids (Peaks 1–6 in chromatogram) and two of the dietary fiber constituents; Sol xyl and Sol glc, while Sol man was significant at *p* < 0.06 (Table 4). Furthermore, for Group 2 of the phenolic acids (Peaks 7–26 in chromatogram), significant positive Pearson correlations (*p* < 0.05) were found with Sol fuc, Sol xyl, Sol man and Sol glc, and negative with Insol UA, Insol ara, and Insol man, respectively (Table 4).

Most of the conjugated phenolics showed negative values for PC1 in the loading plot, thereby indicating a negative relationship with the above mentioned soluble dietary fibers (Sol fuc, Sol xyl, Sol man, Sol glc) (Figure 1a), which was also verified by a negative Pearson correlation between these dietary fiber and both the group Flavonoids (peak B–O in chromatogram) and the group Phenolic acids Derivatives (peak P–Z in chromatogram (Table 4). Furthermore, the Flavonoids also showed significant Pearson correlations with Insol fuc (*p* < 0.05) and with Sol rha, Sol fuc and Sol man at *p* < 0.06.

The rest of the fiber constituents (Insol ara, Sol ara, and Sol gal) showed no relationship to any of the individual phenolics, indicated by their relatively close to zero PC1 values and relatively high positive values on PC2 (Figure 1), which was also verified by the Pearson correlations coefficients (Table 4).

The PCA also clearly depicted the higher content of phenolic acids and soluble fiber constituents (Sol fuc, Sol xyl, Sol man, Sol glc) in leaves from 2018 (Field 3 and Field 4), as compared to leaves from 2017, the latter instead having a higher content of conjugated phenolics (compare Figure 1a with Figure 1b). Leaves from Field 2 showed low levels of insoluble fiber, indicated by their negative PC2 values, while insoluble fiber showed positive PC2 values. Field 1 leaves showed generally large variation of insoluble fiber content. Based on all the dietary fiber constituents and phenolic compounds detected with HPLC, the first two principal components accounted for 48.0 and 17.4% of the variation respectively, adding up about 65% of the variation.

## 4. Discussion

The present study clearly showed that broccoli leaves, today commonly not used as food, have high content of both dietary fiber and phenolic compounds and also that the content of some of the dietary fiber constituents and phenolic compounds co-varied. Broccoli leaves turned out as having high content of compounds regarded as healthy, which make them of interest as potential component for the food industry. Environmental and climate change concern has increased the interest in using edible side-streams of food production for new food products, which also would increase the amount of food available globally. Furthermore, a high content of dietary fiber and phenolic compounds combined is of interest from a health perspective. The co-variation of these compounds might be of specific relevance as a major factor affecting the uptake mechanism in the human intestine. 

Here, we have for the first time, to our knowledge, shown a co-variation in broccoli leaves among content of certain phenolic and dietary fiber, i.e., some of the phenolic acids showed a positive correlation with some of the SDF (Sol fuc, Sol xyl, Sol man and Sol glc; (Table 4)). Three of the mentioned dietary fiber constituents (Sol fuc, Sol xyl and Sol glc) are known as being the main parts of the complex soluble dietary fiber xyloglucan [57]. Previous studies have suggested a possibility that phenolic compounds are bound to the complex dietary fiber xyloglucan [58].

Previous results have indicated that phenolic compounds can be strongly bound to dietary fiber, thereby they should be considered as one collective group, denominated as antioxidant dietary fiber [19,23,59]. However, previous studies have also pointed out that phenolics are a large and diverse group of compounds localized in several parts of the plant cell; in the vacuole, in the chloroplast, in the nuclei, and also in the cell wall [60]. In a study of chicory leaves, the fractions of foliar parenchyma cells were found to have higher concentration of phenolics as compared to vein fractions [61], indicating that cells in the veins with thicker cell walls, constituting of dietary fiber, had lower concentrations of phenolics. The results from the present study showed corresponding results, i.e., in this investigation the dietary fiber constituents of the broccoli leaves present in highest concentration in this investigation (Insol UA, Insol xyl, and Insol glc) showed in general no significant correlation with the analyzed phenolics, and some of both IDF and SDF constituents showed negative correlation with different phenolic groups. Hence, the major part of the phenolics found in this investigation should not be bound to cell walls, i.e., the dietary fiber, but rather be present in other parts of the cells or in cells with thinner cell wall. However, the phenolic compounds are possibly not easily extracted from the fiber matrix with only organic solvent. As described in the materials and method section, we have used methanol extraction following similar procedure as recommended and used in other publications and also by us on other brassica species [48,49,50]. However, the results from the present study indicate that additional phenolic compounds might be present in broccoli leaves not able to be extracted with methods generally adopted and commonly used for phenolics extraction in plants. To be able to evaluate content of all phenolic compounds, and including all cell wall bound phenolic compounds, alternative extraction procedures with a more efficient disruption of the cell wall can be considered, including enzymatic [62], ultrasonic [63] and ultrasonic assisted enzymatic extraction [64,65]. 

Broccoli leaves, with their mean content of TDF at 26%–32% of the DW, have an intermediate content of TDF, as compared to other types of food and vegetables (Table 6). Thus, the content of dietary fiber in broccoli leaves is higher than that in oat brans, carrots and apples, but lower content as compared to onions, cabbage outer leaves, kale leaves and the broccoli florets. This makes broccoli leaves an interesting raw material for food from a health perspective. 

Despite, as discussed above, that content of phenolics might possibly be higher in broccoli leaves than possible to measure with the applied methodology, the levels were found similar as previously reported for kale, and higher as compared to the broccoli florets (Table 7). Thus, from perspective of phenolic content, the broccoli leaves are an interesting component for the food industry. The content of conjugated phenolics in the present study varied between the years, with 10.8–15.2 mg/g DW for 2017 as compared to 6.3–7.5 mg/g DW for 2018. At the same time, the content of phenolic acids did not vary significantly between years, but were approximately 3.6–5.7 mg/g DW. 

Both the content of dietary fiber and phenolics varied between the two years of this study, though the former to somewhat lower degree. This might be due to the different weather conditions during these years, with an exceptionally warm and dry summer in Sweden 2018 (maximum and mean temperature in 2018 were 28.6 °C and 16.4 °C respectively, compared to 20.8 °C and 14.3 °C respectively in 2017, according to Swedish Meteorological and Hydrological Institute (SMHI)). The levels of phenolic compounds in kale, another member of the Brassica family, have been shown to increase when the temperature decreases due to an accumulation of secondary metabolites [67,68]. The amount of phenolic compounds in Brassica also depend on genetic variation (both within and among species) and on environmental factors as well as biotic and abiotic stresses (e.g., insect attacks, light, temperature, nutrients, water, growing conditions, and UV radiation) [51]. Furthermore, in this investigation the broccoli leaves were collected at commercial farms applying crop rotation, i.e., the same fields were not used for broccoli production during the two years. Instead plant materials were collected from fields in the same area both years, resulting in that variation between the two years might also be due to differences between fields. Lastly, water content in the broccoli leaves differed significantly between the years, which also indicate differences in environmental factors which might impact variation in phenolic and dietary fiber between the years. Similar water content have been reported earlier [37,42].

In this investigation we have used the common categorization of the dietary fiber in soluble and insoluble fiber. However, recently it has been questioned if these two categories are sufficient when describing the functionality of the specific type of fiber, and the perceived health effects [4,69]. At present, there is insufficient knowledge of how the individual components of both the dietary fiber and the phenolics influence the various health effects, and also possible interactions between these groups. In addition, the structural diversity of the different fiber, both within a plant, but also depending of the plant species, is likely to influence the digestion of the fiber, and thereby possibly the health effects.

Health beneficial effects from phenolic compound have been suggested to be a result of some phenolics having the opportunity to travel along the intestines to reach the colon, and the gut microbiota, intact [3,26]. Phenolics are suggested to be strongly bound to dietary fiber, and to not be released from the food matrix by mastication, acid pH or human digestive enzymes [70]. The phenolic compounds that travels inside the gastrointestinal tract for a long time together with the dietary fiber might also have the effect that they lower the amounts of reactive oxygen species (e.g., free radicals) in the gastrointestinal tract, which would also be beneficial [19]. Dietary fiber from kale has been shown to bind bile acid and simultaneously release phenolic compounds from the matrix, thus bile acids can increase the bioaccessibility of the phenolic compounds [71], and has also been shown to have a beneficial impact on the cholesterol levels in the blood [72]. In connection to this, the gut microbiota has been shown to be altered by consumption of dietary fiber rich cruciferous vegetable, such as broccoli, cauliflower and cabbage, which could ultimately influence gut metabolism of bioactive food components and host exposure to these beneficial compounds [73]. Phenolic compounds in themselves have been shown to be beneficial for health, e.g., by increasing weight loss in obese mice and humans [74], and also to lower the mortality of some chronic diseases, mainly cardiovascular diseases and cancer [75].

The average daily intake of dietary fiber in most Western countries (15–25 g dietary fiber/day) is low compared with the recommended daily intake of dietary fiber in Europe (20–38 g/day for adults) [4]. The content of dietary fiber found in this investigation in broccoli leaves, 26%–32% of the DW, are in line with earlier studies which showed that the levels of TDF in a mixture of broccoli leaves and stems were approximately 36% [76] with lower amount of fiber in the leaves compared to the stems [42]. Hence, if broccoli side streams are used in every day food products, this will contribute to an increase in total dietary intake of fiber towards the recommended levels, while at the same time lessen the amount of the broccoli plant not used as food. Dietary fiber ingredients can also be used to improve functional properties in, e.g., meat, dairy, and wheat flour-based products [77].

Production of food requires resources such as water, fertilizers, farmland, and energy. Currently, the generated amount of food waste correspond to production on 0.9 million hectares of farmland, release of 3.49 GT carbon dioxide equivalents (CO_2_e), and use of 306 km^3^ of drinkable water [78]. In these calculations, the biomass not harvested but that could be eaten was not included. A more complete use of the agriculturally produced biomass would contribute to an increased productivity with less field waste, which would have a beneficial impact on the global climate. Furthermore, the different side steams from fruit and vegetable production are a readily available resource, and can be used as new food products, but also as a raw material for extraction of valuable compounds [79]. 

In the case of broccoli, the florets only make up 15% of the total biomass of the broccoli plant, while the leaves make up a total of 47%, and stems and roots make up the remaining 38% [37]. Broccoli powder from dried florets, leaves or stalks can be used as a natural food supplement since these powders contain high levels of amino acids and fatty acids and also good physicochemical properties [37,42]. An addition of 10% vegetable powder (carrot, tomato, broccoli florets, and beetroot), has been shown to increase the nutritional and functional attributes in oil-free bread [80]. Broccoli florets increased levels of protein, fat, vitamin E and also antioxidant capacity in these breads. Broccoli leaves and stems have been shown to increase the phenolic content and antioxidant capacity in bread when added in a concentration of 2% (w/w), while still have an overall acceptability [81]. In addition, broccoli leaf powder has been proposed for use in gluten free sponge cake to increase the content of minerals, antioxidant capacity and protein [82], and also to increase the technological and sensory quality of gluten free sponge cake [83]. Hence, with the levels of dietary fiber and phenolic compounds found in broccoli leaves in this study, future food uses of this side stream would be of interest as a food supplement to increase nutritional values. Furthermore, added-value use of the side streams of broccoli leaves contributes to socio-economic and environmental sustainability to the bioeconomy of our modern society.

## 5. Conclusions

Broccoli leaves, a side stream in the broccoli production, contain high levels of dietary fiber and phenolics, comparable with other vegetables currently used as food. Covariation of some SDF of the dietary fiber xyloglucan and phenolic acids may indicate interactions between these components that most likely influence the bioavailability of the phenolics in the human intestine. To further elucidate the relationship between dietary fiber and phenolic acid interactions and effort on bioavailability is of relevance and would require further research combining biology/agronomy and medical expertise. Yearly variation in weather conditions affected the content of conjugated phenolics in the broccoli leaves. Lower levels were recorded during a season with hot and dry weather conditions than in a season with cooler and rainier weather. As highly nutritious and readily available, broccoli leaves is an interesting source to be used as a functional ingredient to increase the nutritional content in different types of food, with resulting potential health benefits. 

## Figures and Tables

**Table 1:** Total content of dietary fiber in broccoli leaves, divided into insoluble (IDF), soluble (SDF) and total (TDF) dietary fiber. TDF was calculated as the sum of IDF and SDF.

	IDF	SDF	TDF
	[% of DW]	[% of DW]	[% of DW]
Field 1 (2017)	30.6 ^b^ ± 4.2	1.9 ^a^ ± 0.4	32.6 ^a^ ± 4.4
Field 2 (2017)	25.0 ^ab^ ± 0.7	1.8 ^a^ ± 0.3	26.8 ^a^ ± 0.5
Field 3 (2018)	25.3 ^ab^ ± 1.5	2.0 ^a^ ± 0.1	27.3 ^a^ ± 1.6
Field 4 (2018)	23.8 ^a^ ± 1.2	2.3 ^a^ ± 0.2	26.0 ^a^ ± 1.3

Data is expressed as mean ± SD (n = 6). Values followed by the same letters do not differ significantly (*p* < 0.05) by using the Tukey post hoc test.

**Table 2:** Content of dietary fiber constituents (Klason lignin and sugar residues) in broccoli leaves from four fields.

	Klason lignin	Insol UA	Insol rha	Insol fuc	Insol ara	Insol xyl	Insol man	Insol gal	Insol glc
Field 1 (2017)	1.8 ^a^ ± 0.6	8.1 ^b^ ± 0.6	0.7 ^a^ ± 0.0	0.2 ^a^ ± 0.0	2.6 ^b^ ± 0.6	2.6 ^a^ ± 0.7	1.0 ^a^ ± 0.0	1.5 ^a^ ± 0.2	12.2 ^a^ ± 1.9
Field 2 (2017)	1.7 ^a^ ± 0.5	7.5 ^ab^ ± 0.4	0.7 ^a^ ± 0.1	0.1 ^a^ ± 0.0	1.6 ^ab^ ± 0.1	1.7 ^a^ ± 0.2	0.9 ^a^ ± 0.0	1.2 ^a^ ± 0.1	9.6 ^a^ ± 0.2
Field 3 (2018)	1.8 ^a^ ± 0.4	7.3 ^ab^ ± 0.4	0.7 ^a^ ± 0.0	0.2 ^a^ ± 0.0	1.1 ^a^ ± 0.1	2.1 ^a^ ± 0.5	0.9 ^a^ ± 0.1	1.3 ^a^ ± 0.1	1.0 ^a^ ± 0.8
Field 4 (2018)	1.6 ^a^ ± 0.4	6.9 ^a^ ± 0.4	0.7 ^a^ ± 0.0	0.2 ^a^ ± 0.0	1.4 ^a^ ± 0.2	1.7 ^a^ ± 0.1	0.9 ^a^ ± 0.1	1.3 ^a^ ± 0.1	9.3 ^a^ ± 0.7
Fields, 2017	1.8 ^a^ ± 0.5	7.8 ^a^ ± 0.6	0.7 ^a^ ± 0.1	0.2 ^a^ ± 0.0	2.1 ^a^ ± 0.7	2.1 ^a^ ± 0.7	1.0 ^a^ ± 0.1	1.4 ^a^ ± 0.2	10.9 ^a^ ± 1.9
Fields, 2018	1.7 ^a^ ± 0.4	7.1 ^b^ ± 0.4	0.7 ^a^ ± 0.0	0.2 ^a^ ± 0.0	1.3 ^b^ ± 0.2	1.9 ^a^ ± 0.4	0.9 ^b^ ± 0.1	1.3 ^a^ ± 0.1	9.6 ^a^ ± 0.8
		**Sol UA**	**Sol rha**	**Sol fuc**	**Sol ara**	**Sol xyl**	**Sol man**	**Sol gal**	**Sol glc**
			**[10^−2^]**	**[10^−2^]**	**[10^−1^]**	**[10^−2^]**	**[10^−1^]**	**[10^−1^]**	**[10^−1^]**
Field 1 (2017)		1.0 ^a^ ± 0.4	5.6 ^a^ ± 0.9	1.3 ^a^ ± 0.6	3.3 ^b^ ± 0.5	2.3 ^ab^ ± 0.1	1.1 ^ab^ ± 0.2	3.1 ^a^ ± 0.4	0.8 ^ab^ ± 0.1
Field 2 (2017)		1.1 ^a^ ± 0.2	4.1 ^a^ ± 0.7	1.1 ^a^ ± 0.6	2.3 ^a^ ± 0.4	1.6 ^a^ ± 0.1	1.0 ^a^ ± 0.2	2.5 ^a^ ± 0.5	0.7 ^a^ ± 0.1
Field 3 (2018)		1.1 ^a^ ± 0.1	5.3 ^a^ ± 1.2	3.1 ^a^ ± 0.8	2.2 ^a^ ± 0.3	3.2 ^b^ ± 0.4	1.4 ^ab^ ± 0.1	2.9 ^a^ ± 0.3	1.1 ^b^ ± 0.1
Field 4 (2018)		1.4 ^a^ ± 0.2	5.9 ^a^ ± 0.7	2.1 ^a^ ± 0.3	2.4 ^ab^ ± 0.2	3.2 ^b^ ± 0.4	1.4 ^b^ ± 0.1	2.8 ^a^ ± 0.1	1.1 ^b^ ± 0.3
Fields, 2017		1.1 ^a^ ± 0.3	4.8 ^a^ ± 0.1	1.2 ^a^ ±0.1	2.8 ^a^ ± 0.5	1.9 ^a^ ± 0.9	1.0 ^a^ ± 0.2	2.8 ^a^ ± 0.4	0.8 ^a^ ± 0.1
Fields, 2018		1.2 ^a^ ± 0.2	4.7 ^a^ ± 1.0	2.1 ^b^ ± 0.1	2.2 ^a^ ± 0.3	2.4 ^b^ ± 0.7	1.2 ^b^ ± 0.2	2.7 ^a^ ± 0.4	0.9 ^b^ ± 0.1

Values are mean [% of DW] ± SD. Values followed by the same letters do not differ significantly at *p* < 0.05 by using the Tukey post hoc test. From each field, three plants were analysed in duplicates. Leaves, 2017 and Leaves, 2018 are the total amount of the constituent from the two fields from each year respectively. Insol: insoluble. Sol: Soluble. The sugar residues are annotated UA: uronic acid. rha: rhamnose. fuc: fucose. ara: arabinose. xyl: xylose. man: mannose. gal: galactose. glc: glucose.

**Table 3:** Content of phenolic compounds in broccoli leaves.

	Conjugated Phenolics	Phenolic Acids (after Hydrolysis)
	[mg/g DW]	[mg/g DW]
Field 1, 2017	10.8 ^ab^ ± 1.8	4.4 ^a^ ± 1.7
Field 2, 2017	15.2 ^b^ ± 4.8	3.6 ^a^ ± 1.0
Field 3, 2018	6.3 ^a^ ± 1.1	5.3 ^a^ ± 1.2
Field 4, 2018	7.5 ^a^ ± 0.6	5.7 ^a^ ± 1.1

Values shown are the mean of three replicates [% of DW] ± SD. Values followed by the same letters do not differ significantly at *p* < 0.05 by using the Tukey post hoc test. For Field 4, one replicate out of nine were removed due to experimental error.

**Table 4:** Pearson correlation coefficients among dietary fiber constituents and groups of phenolic compounds.

	Group 1	Group 2	Phenolic Acid Derivatives	Flavonoids	Colour Legend	
Klason lignin	−0.41	−0.05	0.04	0.02		
Insol UA	−0.47	−0.59	0.36	0.11		
Insol rha	−0.18	−0.11	0.01	−0.18		
Insol fuc	0.14	0.25	−0.43	−0.61	*p*−value	
Insol ara	−0.42	−0,65	0.44	0.16	> 0.05	
Insol xyl	0.08	−0.13	−0.04	−0.21	0.05–0.01	
Insol man	−0.36	−0.59	0.48	0.27	0.01–0.001	
Insol gal	−0.12	−0.22	0.10	−0.23	<0.001	
Insol glc	−0.18	−0.33	0.16	−0.06		
Sol UA	0.54	0.33	0.09	−0.14		
Sol rha	0.47	0.42	−0.44	−0,57		
Sol fuc	0.39	0.72	−0.76	−0.56		
Sol ara	−0.29	−0.46	0.23	0.00		
Sol xyl	0.69	0.83	−0.72	−0.88		
Sol man	0.56	0.71	−0.73	−0.56		
Sol gal	−0.02	0.02	−0.21	−0.35		
Sol glc	0.74	0.84	−0.72	−0.66		

Flavonoids: Peaks B-O from the chromatogram of methanol extract (conjugated phenolics). Phenolic acid derivatives: Peaks P-Z from the chromatogram of methanol extract (conjugated phenolics) (Figure S1). Group 1: Peaks 1–6 in chromatogram after alkaline hydrolysis (phenolic acids). Group 2: Peaks 7–26 in the chromatogram after the alkaline hydrolysis (phenolic acids). The scatter plots with significant *p*-values can be found in Figures S2–S6 in Supplementary Materials.

**Table 5:** Tentative identification of phenolic compounds in methanol extract of broccoli leaves

Peak ID	Ret.time [min]	DAD [nm]	MS Scan(−)	MS Sim	Suggested Identification
A	10.37	326, 299	353, 1138.8	353	caffeoylquinic acid (chlorogenic acid)
B	13.06	341, 318	1157, 609		K-3-O-(sinapoyl)-sophoroside-7-O-diglucoside
C	13.29	346	771, 1159		K-3-O-(sinapoyl)-sophoroside-7-O-glucoside
D	13.66	333	1538		isorhamnetin-3-O-(disinapoyl)-sophorotrioside-7-O-diglucoside
E	14.03	327	963, 1125	963.4	K-3-O-(methoxycaffeoyl)-sophoroside-7-O-diglucoside
F	14.267	333	269	933	Unidentified phenolic compound
G	14.35	334	933, 1097	933.4	K-3-O-caffeoyl-sophoroside-7-O-diglucoside
H	14.56	340	993		Q-3-O-(sinapoyl)-sophoroside-7-O-glucoside
I	14.74	333	963	963.4	K-3-O-hydroxyferuloyl-sophoroside-7-O-glucoside
J	14.88	330	1139, 1175	933.4	K-3-O-caffeoyl-sophoroside-7-O-glucoside
K	15.05	332	1139		K-3-O-sinapoyl-sophorotrioside-7glucoside
L	15.23	336	977	977.5	K-3-O(sinapoyl)-sophoroside-7glucoside
M	15.35	339	1109	1109.5	K-3-O(feruloyl)sophoroside-7-O-diglucoside
N	15.60	332	947	947.5	K-3-O(feruloyl)sophoroside-7-O-glucoside
O	15.70	269, 341	428.2, 195, 425		Unidentified phenolic compound
P	20.62	330	731, 975, 1123, 1367		Unidentified phenolic compound
Q	20.97	332	771, 1507		K-3-O(disinapoyl)sophorotrioside-7-O-diglucoside
R	20.26				Unidentified phenolic compound
S	21.33	331	1538		isorhamnetin-3-O-(disinapoyl)-sophorotrioside-7-O-diglucoside
T	21.62	329	1316		Q-3-O(disinapoyl)sophorotrioside7-O-diglucoside
U	22.71	330	753	1402	disinapoyl-diglucoside
V	23.07	327	723		sinapoyl-feruloyl- diglucoside
W	23.33	326	693		diferuloyl-diglucoside
X	24.16	324	959		trisinapoyl-diglucoside
Y	24.48	325	617, 653, 1236		phenolic acid derivate

K stands for kaempferol, Q stands for quercetin. All peaks were not detectable in all samples. For MS Scan (−), the main fragments are reported.

**Table 6:** Comparing levels of total dietary fiber in food.

Sample	Mean [% of DW]	Reference
Potex	80.4	[44]
Onion	47.2	[16]
Kale leaves	42.7	[15]
Cabbage outer leaves	40.9	[17]
Broccoli florets	36.0	[16]
Broccoli leaves	26–32	[present study]
Cauliflower (curd)	29.7	[16]
Carrot	24.1	[44]
Oat bran	18.4	[44]
Apple	17.9	[44]
Green peas	16.7	[44]
Rye bread	10.3	[44]
White bread	4.6	[44]

**Table 7:** Comparing content of phenolic compounds by methanol extraction.

Sample	mg/g DW	Reference
Broccoli leaves, 2017	10.8–15.2	[present study]
Kale leaves	10.6	[33]
Broccoli leaves, 2018	6.3–7.5	[present study]
Broccoli florets	1.7–2.2	[66]

## Supplementary Materials

The following are available online at https://www.mdpi.com/1660-4601/17/7/2406/s1, Figure S1: examples of chromatograms, Figure S2: correlation between the group phenolic acids (methanol extraction) and dietary fiber constituents, Figure S3: correlation between the group flavonoids (methanol extraction) and dietary fiber constituents, Figure S4: correlation between Group 1 (Peaks 1–6 in the chromatogram for alkaline hydrolysis) and the constituents of dietary fiber, Figure S5: correlation between phenolic Group 2 (Peaks 7–26 in the chromatogram for the alkaline hydrolysis) and the insoluble dietary fiber, Figure S6: correlation between phenolic Group 2 (Peaks 7–26 in chromatogram for the alkaline hydrolysis) and the soluble constituents of dietary fiber. Table S1: suggested identification for the peaks in alkaline hydrolysis of broccoli leaves. Figure S7: examples of HPLC and MS spectra for phenolic compounds in methanol extract. Figure S8: examples of HPLC and MS spectra for phenolic compounds after alkaline hydrolysis

## References

[1] Oyebode O., Gordon-Dseagu V., Walker A., Mindell J.S. (2014). Fruit and vegetable consumption and all-cause, cancer and CVD mortality: Analysis of Health Survey for England data. J. Epidemiol. Community Health.

[2] Liu R.H. (2013). Health-promoting components of fruits and vegetables in the diet. Adv. Nutr..

[3] Williamson G. (2017). The role of polyphenols in modern nutrition. Nutr. Bull..

[4] Stephen A.M., Champ M.M.-J., Cloran S.J., Fleith M., van Lieshout L., Mejborn H., Burley V.J. (2017). Dietary fibre in Europe: Current state of knowledge on definitions, sources, recommendations, intakes and relationships to health. Nutr. Res. Rev..

[5] Kim Y., Je Y. (2016). Dietary fibre intake and mortality from cardiovascular disease and all cancers: A meta-analysis of prospective cohort studies. Arch. Cardiovasc. Dis..

[6] (2008). Codex Alimentarius Guidelines on Nutrition Labelling CAC/GL2-1985 as Last Revision in 2015.

[7] Hassan F.A., Ismail A., Hamid A.A., Azlan A., Al-sheraji S.H. (2011). Characterisation of fibre-rich powder and antioxidant capacity of Mangifera pajang K. fruit peels. Food Chem..

[8] Mandimika T., Paturi G., De Guzman C.E., Butts C.A., Nones K., Monro J.A., Butler R.C., Joyce N.I., Mishra S., Ansell J. (2012). Effects of dietary broccoli fibre and corn oil on serum lipids, faecal bile acid excretion and hepatic gene expression in rats. Food Chem..

[9] Mackie A., Bajka B., Rigby N. (2016). Roles for dietary fibre in the upper GI tract: The importance of viscosity. Food Res. Int..

[10] Wrick K., Robertson J., Vansoest P., Lewis B., Rivers J., Roe D., Hackler L. (1983). The influence of dietary fiber source on human intestinal transit and stool output. J. Nutr..

[11] Makki K., Deehan E.C., Walter J., Bäckhed F. (2018). The impact of dietary fiber on gut microbiota in host health and disease. Cell Host Microbe.

[12] Desai M.S., Seekatz A.M., Koropatkin N.M., Kamada N., Hickey C.A., Wolter M., Pudlo N.A., Kitamoto S., Terrapon N., Muller A. (2016). A dietary fiber-deprived gut microbiota degrades the colonic mucus barrier and enhances pathogen susceptibility. Cell.

[13] Paturi G., Butts C., Monro J., Nones K., Martell S., Butler R., Sutherland J. (2010). Cecal and colonic responses in rats fed 5 or 30% corn oil diets containing either 7.5% broccoli dietary fiber or microcrystalline cellulose. J. Agric. Food Chem..

[14] McKee L.H., Latner T.A. (2000). Underutilized sources of dietary fiber: A review. Plant Food Hum. Nutr..

[15] Thavarajah D., Siva N., Johnson N., McGee R., Thavarajah P. (2019). Effect of cover crops on the yield and nutrient concentration of organic kale (Brassica oleracea L. var. acephala). Sci. Rep..

[16] Kalala G., Kambashi B., Everaert N., Beckers Y., Richel A., Pachikian B., Neyrinck A.M., Delzenne N.M., Bindelle J. (2018). Characterization of fructans and dietary fibre profiles in raw and steamed vegetables. Int. J. Food Sci. Nutr..

[17] Tanongkankit Y., Chiewchan N., Devahastin S. (2012). Physicochemical property changes of cabbage outer leaves upon preparation into functional dietary fiber powder. Food Bioprod. Process..

[18] Edwards C.A., Havlik J., Cong W., Mullen W., Preston T., Morrison D.J., Combet E. (2017). Polyphenols and health: Interactions between fibre, plant polyphenols and the gut microbiota. Nutr. Bull..

[19] Saura-Calixto F. (2011). Dietary fiber as a carrier of dietary antioxidants: An essential physiological function. J. Agric. Food Chem..

[20] Quirós-Sauceda A.E., Palafox-Carlos H., Sáyago-Ayerdi S.G., Ayala-Zavala J.F., Bello-Perez L.A., Álvarez-Parrilla E., de la Rosa L.A., González-Córdova A.F., González-Aguilar G.A. (2014). Dietary fiber and phenolic compounds as functional ingredients: Interaction and possible effect after ingestion. Food Funct..

[21] Phan A.D.T., Netzel G., Wang D., Flanagan B.M., D’Arcy B.R., Gidley M.J. (2015). Binding of dietary polyphenols to cellulose: Structural and nutritional aspects. Food Chem..

[22] Gonzalez-Aguilar G.A., Blancas-Benitez F.J., Sayago-Ayerdi S.G. (2017). Polyphenols associated with dietary fibers in plant foods: Molecular interactions and bioaccessibility. Curr. Opin. Food Sci..

[23] Perez-Jimenez J., Serrano J., Tabernero M., Arranz S., Diaz-Rubio M.E., Garcia-Diz L., Goni I., Saura-Calixto F. (2009). Bioavailability of phenolic antioxidants associated with dietary fiber: Plasma antioxidant capacity after acute and long-term intake in humans. Plant Food Hum. Nutr..

[24] Palafox-Carlos H., Ayala-Zavala J.F., Gonzalez-Aguilar G.A. (2011). The Role of dietary fiber in the bioaccessibility and bioavailability of fruit and vegetable antioxidants. J. Food Sci..

[25] Shahidi F., Naczk M. (2004). Phenolics in Food and Nutraceuticals.

[26] Selma M.V., Espín J.C., Tomás-Barberán F.A. (2009). Interaction between phenolics and gut microbiota: Role in human health. J. Agric. Food Chem..

[27] Shi H., Noguchi N., Pokorny J., Yanishlieva N., Gordon M. (2001). Introducing natural antioxidants. Antioxidants in Food.

[28] Wang S., Melnyk J.P., Tsao R., Marcone M.F. (2011). How natural dietary antioxidants in fruits, vegetables and legumes promote vascular health. Food Res. Int..

[29] Kyle J.A.M., Sharp L., Little J., Duthie G.G., McNeill G. (2010). Dietary flavonoid intake and colorectal cancer: A case-control study. Br. J. Nutr..

[30] Kasprzak K., Oniszczuk T., Wojtowicz A., Waksmundzka-Hajnos M., Olech M., Nowak R., Polak R., Oniszczuk A. (2018). Phenolic acid content and antioxidant properties of extruded corn snacks enriched with kale. J. Anal. Methods Chem..

[31] Kumar B.R. (2017). Application of HPLC and ESI-MS techniques in the analysis of phenolic acids and flavonoids from green leafy vegetables (GLVs). J. Pharm. Anal..

[32] Lin L.-Z., Harnly J.M. (2009). Identification of the phenolic components of collard greens, kale, and Chinese broccoli. J. Agric. Food Chem..

[33] Olsen H., Aaby K., Borge G.I.A. (2009). Characterization and quantification of flavonoids and hydroxycinnamic acids in curly kale (Brassica oleracea L. Convar. acephala Var. sabellica) by HPLC-DAD-ESI-MSn. J. Agric. Food Chem..

[34] Schmidt S., Zietz M., Schreiner M., Rohn S., Kroh L.W., Krumbein A. (2010). Identification of complex, naturally occurring flavonoid glycosides in kale (Brassica oleracea var. sabellica) by high-performance liquid chromatography diode-array detection/electrospray ionization multi-stage mass spectrometry. Rapid Commun. Mass Spectrom..

[35] Vasanthi H.R., Mukherjee S., Das D.K. (2009). Potential health benefits of broccoli—A chemico-biological overview. Mini Rev. Med. Chem..

[36] Ares A.M., Nozal M.J., Bernal J. (2013). Extraction, chemical characterization and biological activity determination of broccoli health promoting compounds. J. Chromatogr. A.

[37] Liu M., Zhang L., Ser S.L., Cumming J.R., Ku K.-M. (2018). Comparative phytonutrient analysis of broccoli by-products: The potentials for broccoli by-product utilization. Molecules.

[38] Bhandari S.R., Kwak J.-H. (2014). Seasonal variation in phytochemicals and antioxidant activities in different tissues of various Broccoli cultivars. AJB.

[39] Bhandari S.R., Kwak J.-H. (2015). Seasonal variation in contents of sugars in different parts of broccoli. Korean J. Hortic. Sci. Technol..

[40] Schäfer J., Stanojlovic L., Trierweiler B., Bunzel M. (2017). Storage related changes of cell wall based dietary fiber components of broccoli (Brassica oleracea var. italica) stems. Food Res. Int..

[41] Dominguez-Perles R., Carmen Martinez-Ballesta M., Carvajal M., Garcia-Viguera C., Moreno D.A. (2010). Broccoli-derived by-products-A promising source of bioactive ingredients. J. Food Sci..

[42] Campas-Baypoli O.N., Sanchez-Machado D.I., Bueno-Solano C., Nunez-Gastelum J.A., Reyes-Moreno C., Lopez-Cervantes J. (2009). Biochemical composition and physicochemical properties of broccoli flours. Int. J. Food Sci. Nutr..

[43] British Research Leads to UK-Wide Launch of Beneforté Broccoli. https://quadram.ac.uk/beneforte_uk_wide/.

[44] Theander O., Aman P., Westerlund E., Andersson R., Petersson D. (1995). Total dietary fiber determined as neutral sugar residues, uronic acid residues, and Klason Lignin (The Uppsala method): Collaborative study. J. AOAC Int..

[45] Andersson A.A.M., Merker A., Nilsson P., Sørensen H., Åman P. (1999). Chemical composition of the potential new oilseed crops Barbarea vulgaris, Barbarea verna and Lepidium campestre. J. Sci. Food Agric..

[46] Lin L.-Z., Chen P., Harnly J.M. (2008). New phenolic components and chromatographic profiles of green and fermented teas. J. Agric. Food Chem..

[47] Jessica N., Kerstin O., Gabriele E., Jimmy E., Marie O., Margareta N. (2006). Åkesson Björn Variation in the content of glucosinolates, hydroxycinnamic acids, carotenoids, total antioxidant capacity and low-molecular-weight carbohydrates in Brassica vegetables. J. Sci. Food Agric..

[48] Harbaum B., Hubbermann E.M., Zhu Z., Schwarz K. (2008). Free and bound phenolic compounds in leaves of pak choi (Brassica campestris L. ssp. chinensis var. communis) and Chinese leaf mustard (Brassica juncea Coss). Food Chem..

[49] Benincasa P., Galieni A., Manetta A.C., Pace R., Guiducci M., Pisante M., Stagnari F. (2015). Phenolic compounds in grains, sprouts and wheatgrass of hulled and non-hulled wheat species. J. Sci. Food Agric..

[50] Klopsch R., Baldermann S., Voss A., Rohn S., Schreiner M., Neugart S. (2018). Bread enriched with legume microgreens and leaves-ontogenetic and baking-driven changes in the profile of secondary plant metabolites. Front. Chem..

[51] Cartea M.E., Francisco M., Soengas P., Velasco P. (2011). Phenolic compounds in brassica vegetables. Molecules.

[52] RStudio Team (2015). RStudio: Integrated Development for R.

[53] Johansson E., Hussain A., Kuktaite R., Andersson S.C., Olsson M.E. (2014). Contribution of organically grown crops to human health. Int. J. Environ. Res. Public Health.

[54] Johansson E., Branlard G., Cuniberti M., Flagella Z., Hüsken A., Nurit E., Peña R.J., Sissons M., Vazquez D., Igrejas G., Ikeda T.M., Guzmán C. (2020). Genotypic and environmental effects on wheat technological and nutritional quality. Wheat Quality for Improving Processing and Human Health.

[55] Vagiri M., Ekholm A., Öberg E., Johansson E., Andersson S.C., Rumpunen K. (2013). Phenols and ascorbic acid in black currants (Ribes nigrum L.): Variation due to genotype, location, and year. J. Agric. Food Chem..

[56] Moreira-Ascarrunz S.D., Larsson H., Prieto-Linde M.L., Johansson E. (2016). Mineral nutritional yield and nutrient density of locally adapted wheat genotypes under organic production. Foods.

[57] Smith B.G., Melton L.D. (2013). Plant cell wall polysaccharides. Food Carbohydrate Chemistry.

[58] Dobson C.C., Mottawea W., Rodrigue A., Buzati Pereira B.L., Hammami R., Power K.A., Bordenave N., Ferreira I.C.F.R., Barros L. (2019). Impact of molecular interactions with phenolic compounds on food polysaccharides functionality. Advances in Food and Nutrition Research.

[59] Saura-Calixto F. (1998). Antioxidant dietary fiber product: A new concept and a potential food ingredient. J. Agric. Food Chem..

[60] Agati G., Azzarello E., Pollastri S., Tattini M. (2012). Flavonoids as antioxidants in plants: Location and functional significance. Plant Sci..

[61] Goupy P.M., Varoquaux P.J.A., Nicolas J.J., Macheix J.J. (1990). Identification and localization of hydroxycinnamoyl and flavonol derivatives from endive (Cichorium endivia L. cv. Geante Maraichere) leaves. J. Agric. Food Chem..

[62] Puri M., Sharma D., Barrow C.J. (2012). Enzyme-assisted extraction of bioactives from plants. Trends Biotechnol..

[63] Virot M., Tomao V., Le Bourvellec C., Renard C.M.C.G., Chemat F. (2010). Towards the industrial production of antioxidants from food processing by-products with ultrasound-assisted extraction. Ultrason. Sonochem..

[64] Wu H., Zhu J., Yang L., Wang R., Wang C. (2015). Ultrasonic-assisted enzymatic extraction of phenolics from broccoli (Brassica oleracea L. var. italica) inflorescences and evaluation of antioxidant activity invitro. Food Sci. Technol. Int..

[65] Wu H., Zhu J.X., Yang L., Wang R., Wang C.R. (2014). Optimization of ultrasonic-assisted enzymatic extraction of phenolics from broccoli inflorescences. Advanced Engineering and Technology.

[66] Torres-Contreras A.M., Nair V., Cisneros-Zevallos L., Jacobo-Velázquez D.A. (2017). Stability of bioactive compounds in broccoli as affected by cutting styles and storage time. Molecules.

[67] Neugart S., Kläring H.-P., Zietz M., Schreiner M., Rohn S., Kroh L.W., Krumbein A. (2012). The effect of temperature and radiation on flavonol aglycones and flavonol glycosides of kale (Brassica oleracea var. sabellica). Food Chem..

[68] Neugart S., Baldermann S., Hanschen F.S., Klopsch R., Wiesner-Reinhold M., Schreiner M. (2018). The intrinsic quality of brassicaceous vegetables: How secondary plant metabolites are affected by genetic, environmental, and agronomic factors. Sci. Hortic..

[69] Gidley M.J., Yakubov G.E. (2019). Functional categorisation of dietary fibre in foods: Beyond ‘soluble’ vs ‘insoluble. ’ Trends Food Sci. Technol..

[70] Pérez-Jiménez J., Díaz-Rubio M.E., Saura-Calixto F. (2013). Non-extractable polyphenols, a major dietary antioxidant: Occurrence, metabolic fate and health effects. Nutr. Res. Rev..

[71] Yang I., Jayaprakasha G.K., Patil B. (2018). In vitro digestion with bile acids enhances the bioaccessibility of kale polyphenols. Food Funct..

[72] Kahlon T.S., Chapman M.H., Smith G.E. (2007). In vitro binding of bile acids by spinach, kale, brussels sprouts, broccoli, mustard greens, green bell pepper, cabbage and collards. Food Chem..

[73] Li F., Hullar M.A.J., Schwarz Y., Lampe J.W. (2009). Human gut bacterial communities are altered by addition of cruciferous vegetables to a controlled fruit- and vegetable-free diet. J. Nutr..

[74] Raiola A., Errico A., Petruk G., Monti D.M., Barone A., Rigano M.M. (2018). Bioactive compounds in brassicaceae vegetables with a role in the prevention of chronic diseases. Molecules.

[75] Ivey K.L., Hodgson J.M., Croft K.D., Lewis J.R., Prince R.L. (2015). Flavonoid intake and all-cause mortality. Am. J. Clin. Nutr..

[76] Shi M., Hlaing M.M., Ying D., Ye J., Sanguansri L., Augustin M.A. (2019). New food ingredients from broccoli by-products: Physical, chemical and technological properties. Int. J. Food Sci. Technol..

[77] Yang Y., Ma S., Wang X., Zheng X. (2017). Modification and application of dietary fiber in foods. J. Chem..

[78] FAO (2014). Mitigation of Food Wastage – Societal Costs and Benefits.

[79] Schieber A., Doyle M.P., Klaenhammer T.R. (2017). Side streams of plant food processing as a source of valuable compounds: Selected examples. Annual Review of Food Science and Technology.

[80] Ranawana V., Campbell F., Bestwick C., Nicol P., Milne L., Duthie G., Raikos V. (2016). Breads fortified with freeze-dried vegetables: Quality and nutritional attributes. Part II: Breads not containing oil as an ingredient. Foods.

[81] Lafarga T., Gallagher E., Bademunt A., Viñas I., Bobo G., Villaró S., Aguiló-Aguayo I. (2019). Bioaccessibility, physicochemical, sensorial, and nutritional characteristics of bread containing broccoli co-products. J. Food Process. Preserv..

[82] Drabińska N., Ciska E., Szmatowicz B., Krupa-Kozak U. (2018). Broccoli by-products improve the nutraceutical potential of gluten-free mini sponge cakes. Food Chem..

[83] Krupa-Kozak U., Drabińska N., Rosell C.M., Fadda C., Anders A., Jeliński T., Ostaszyk A. (2019). Broccoli leaf powder as an attractive by-product ingredient: Effect on batter behaviour, technological properties and sensory quality of gluten-free mini sponge cake. Int. J. Food Sci. Technol..

