# Supplementary material for: Side Streams of Broccoli Leaves: A Climate Smart and Healthy Food Ingredient
Source: Int J Environ Res Public Health. 2020 Apr 1;17(7):2406. doi: 10.3390/ijerph17072406 (PMC7178181; doi:10.3390/ijerph17072406)
Supplement: Supplementary file 1 [file ijerph-17-02406-s001.pdf]

Supplementary materials

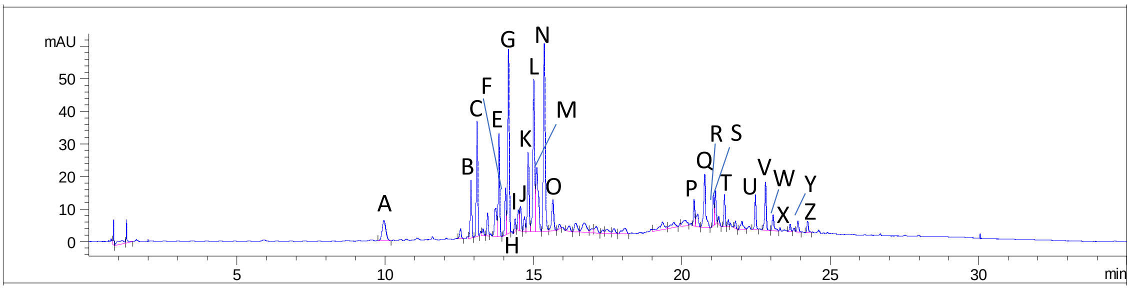

(a)

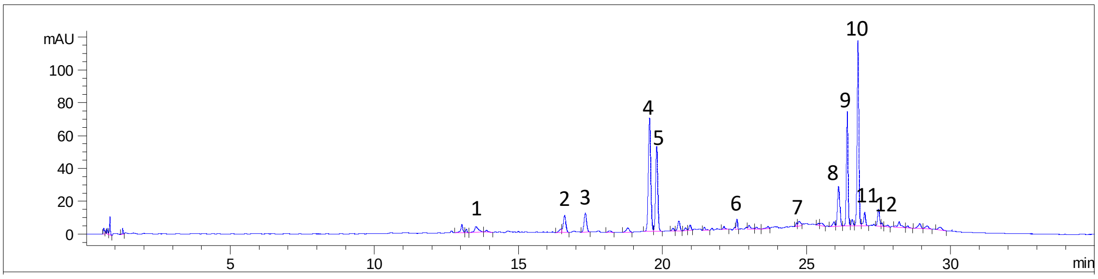

(b)

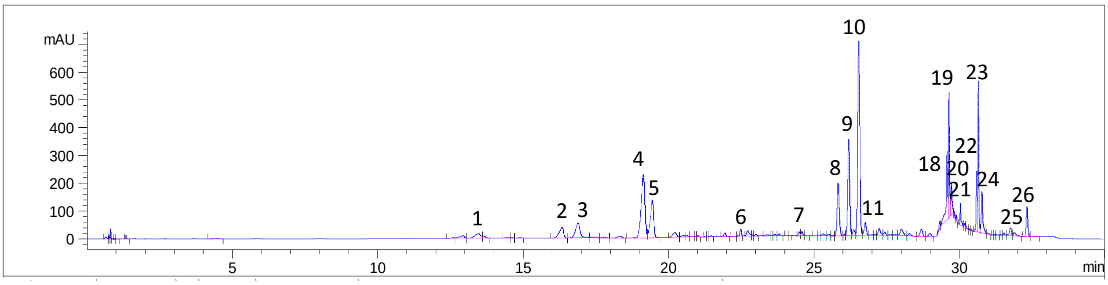

(c)

Figure S1: Examples of chromatogram. (a) Methanol extract of broccoli leaves. Suggested identification can be found in Table S1 (b) Alkaline hydrolysis of broccoli leaves, 2017 (c) Alkaline hydrolysis of broccoli leaves, 2018. Suggested identification for peaks in alkaline hydrolysis can be found in

Table S1.

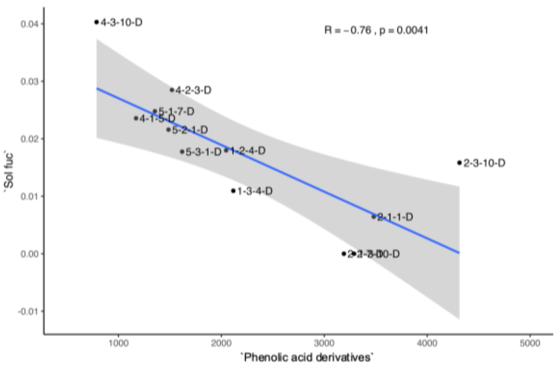

(a)

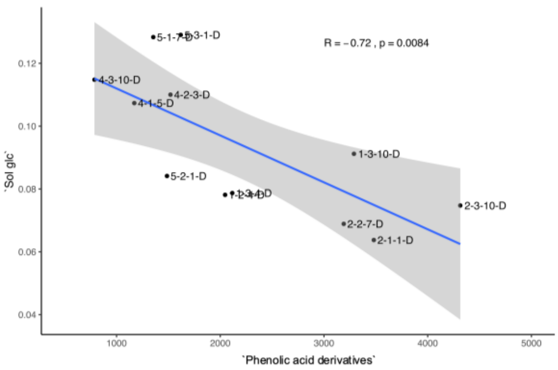

(b)

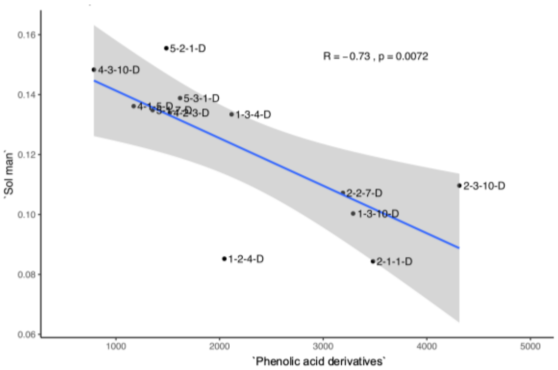

(c)

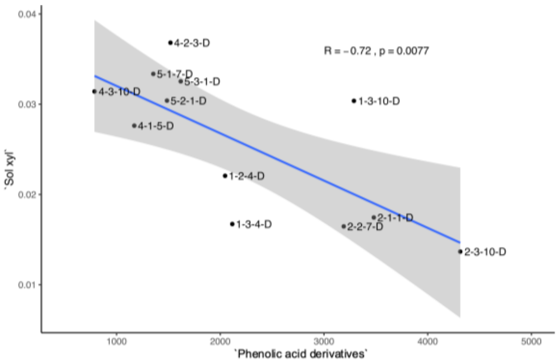

(d)

Figure S2: Correlation between dietary fibre constituents and the group Phenolic acid derivatives from methanol extraction. Only correlations that were significant ($p < 0.05$) are shown. The samples are named with the fashion Year – Field – Square – Part, with D being broccoli leaf. (a) Sol fuc and the group Phenolic acid derivatives with a negative correlation. (b) Sol glc and the group Phenolic acid derivatives with a negative correlation. (c) Sol man and the group Phenolic acid derivatives with a negative correlation (d) Sol xyl and the group Phenolic acid derivatives with a negative correlation.

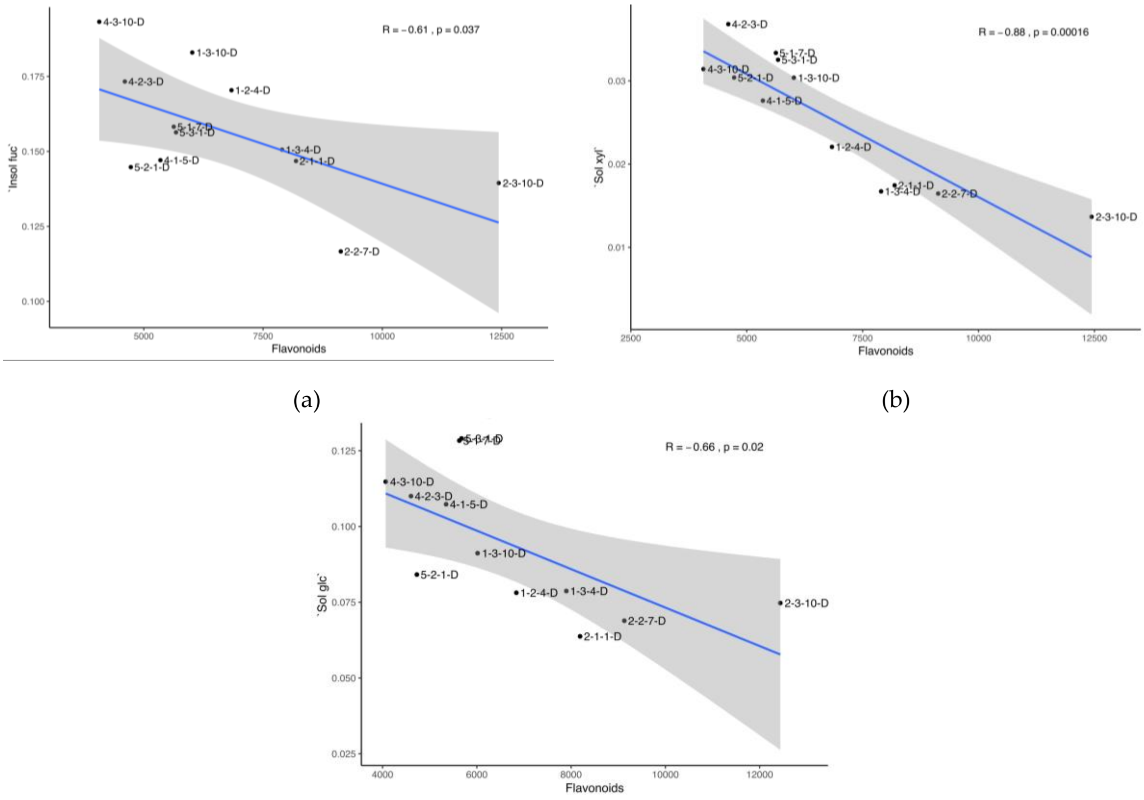

(a) (b)

(c)

Figure S3: Correlation between dietary fibre constituents and group Flavonoids from methanol extraction. Only correlations that were significant ($p < 0.05$) are shown. The samples are named with the fashion Year – Field – Square – Part, with D being broccoli leaf (a) Insol fuc and Flavonoids, with a negative correlation. (b) Sol xyl and Flavonoids with a negative correlation (c) Sol glc, with a negative correlation.

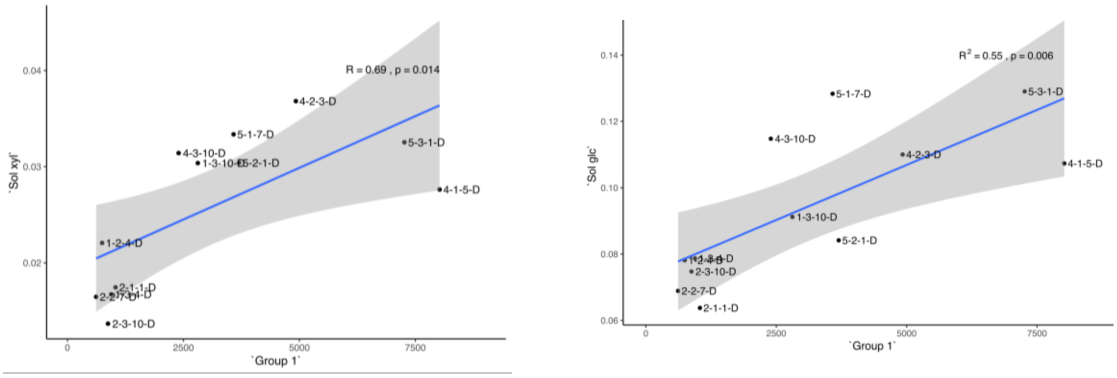

(a) (b)

Figure S4: Correlation between the Group 1 (peak 1-6 in the chromatogram from alkaline hydrolysis) and the constituents of dietary fibre. Only correlations that were significant ($p < 0.05$) are shown. The samples are named with the fashion Year – Field – Square – Part, with D being broccoli leaf. (a) Sol xyl and Group 1, with a positive correlation, (b) Sol glc with Group 1, with close to positive correlation.

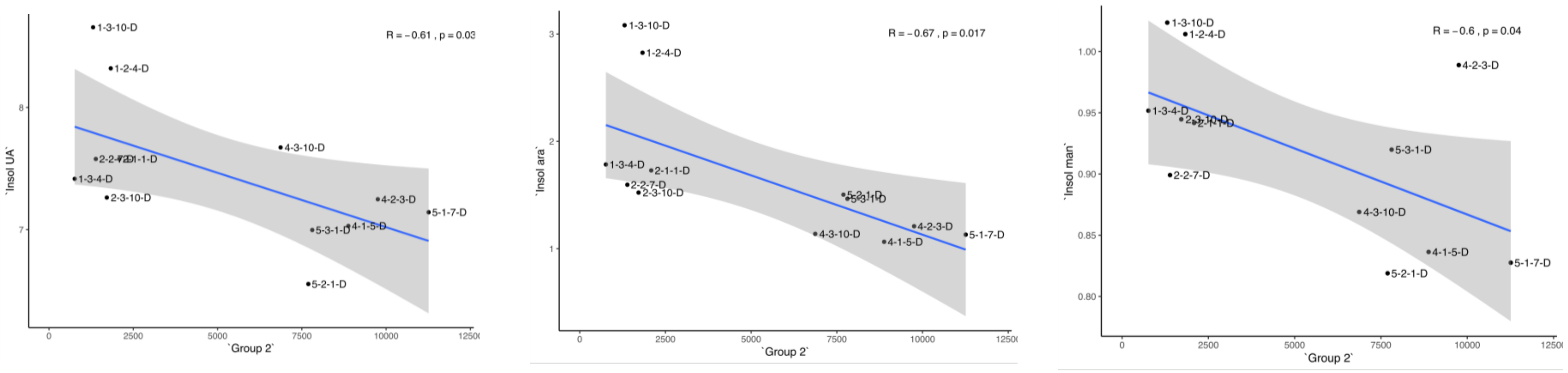

(a) (b) (c)

Figure S5**:** Correlation between phenolic Group 2 (peak 7-26 in the chromatogram from the alkaline hydrolysis) and the insoluble constituents of dietary fibre. Only correlations what were significant ($p < 0.05$) are shown. The samples are named with the fashion Year – Field – Square – Part, with D being broccoli leaf. (a) Insol UA and Group 2 with negative correlation, (b) Insol ara and Group 2 with negative correlation, (c) Insol man and Group 2 with negative correlation

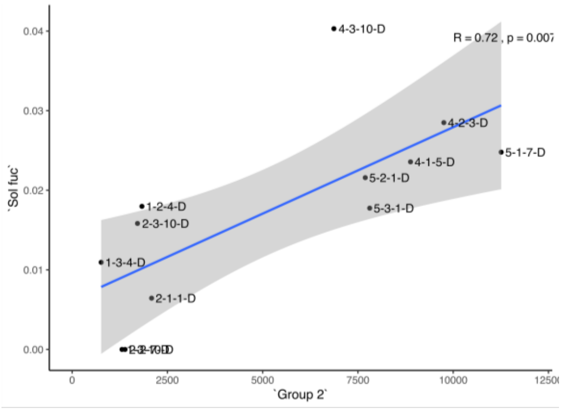

(a)

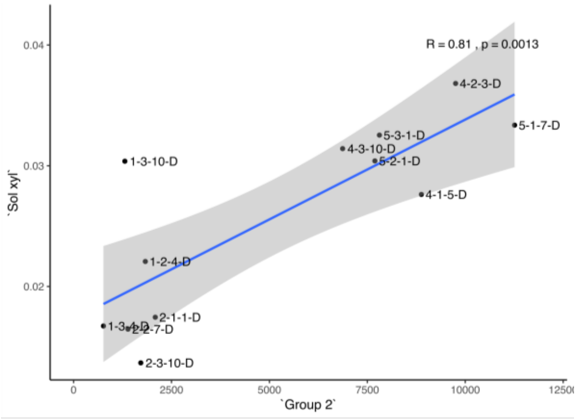

(b)

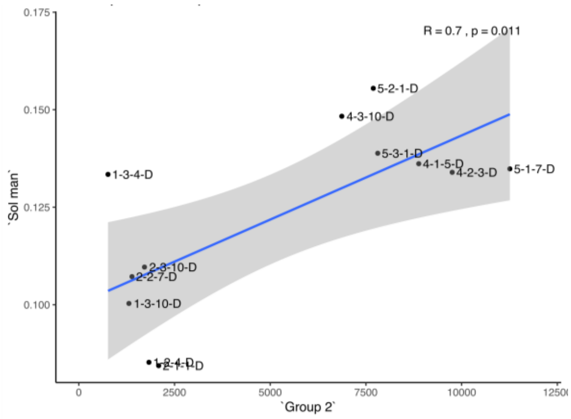

(c)

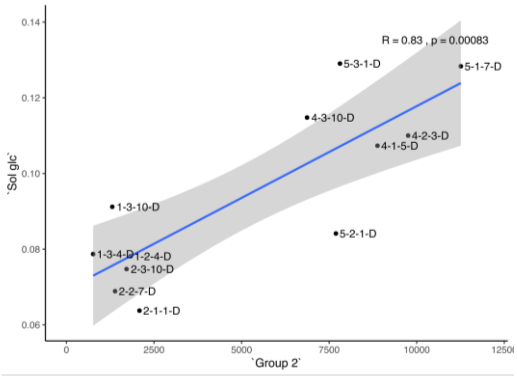

(d)

Figure S6**:** Correlation between phenolic Group 2 (peak 7-26 in the chromatogram from the alkaline hydrolysis) and the soluble constituents of dietary fibre. Only correlations that were significant ($p < 0.05$) are shown. The samples are named with the fashion Year – Field – Square – Part, with D being broccoli leaf. (a) Sol fuc and Group 2 with positive correlation, (b) Sol xyl and Group 2 with positive correlation, (c) Sol man and Group 2 with positive correlation, (d) Sol glc and Group 2 with positive correlation

Table S1: Suggested identification for the peaks in alkaline hydrolysis of broccoli leaves.

Peak ID	Ret.time [min]	DAD [nm]	MS SIM	Suggested identification
1	13.484	288		Unidentified phenolic compound
2	16.379	286	163.0	p-coumaric acid
3	16.913	309		Unidentified phenolic compound
4	19.163	323	193.0	ferulic acid
5	19.471	324	223	sinapic acid
6	21.955	261, 320, 366		Unidentified phenolic compound
7	24.608	324		Unidentified phenolic compound
8	25.85	311		coumaric acid (+methyl group)
9	26.217	326		sinapic acid (+methyl group)
10	26.563	324	163.0, 193.0, 353.0, 137.0	ferulic acid (+methyl group)
11	26.789	319		Unidentified phenolic compound
12	27.262	283		Unidentified phenolic compound
13	28.042	282		Unidentified phenolic compound
14	28.306	282		Unidentified phenolic compound
15	28.724	282		Unidentified phenolic compound
16	29.011	324	169.0, 609.0, 353.0	gallic acid, rutin, chlorogenic acid
17	29.337	297		Unidentified phenolic compound
18	29.584	288		Unidentified phenolic compound
19	29.648	305	193.0, 163.0, 353.0, 289.0	ferulic acid, p-coumaric acid, chlorogenic acid
20	29.902	306		Unidentified phenolic compound
21	30.044	277		Unidentified phenolic compound
23	30.793	307, 403		Unidentified phenolic compound
24	31.547	247, 322		Unidentified phenolic compound
25	31.906	284, 403		Degraded chlorophyll
26	32.341	277		Unidentified phenolic compound

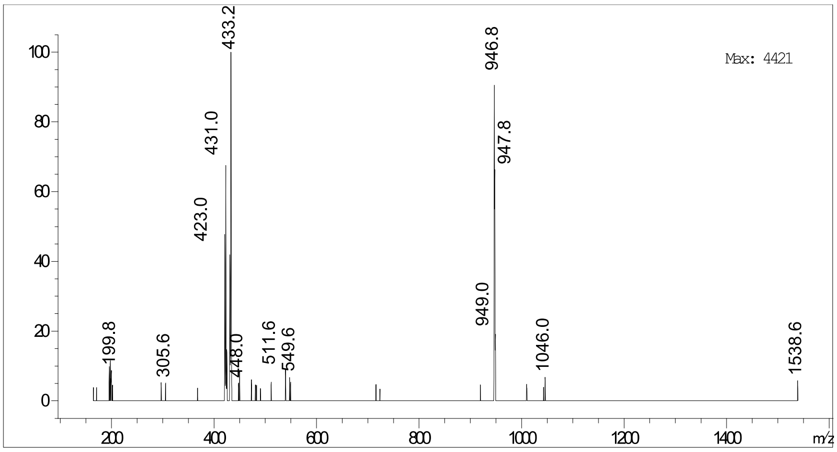

(a)

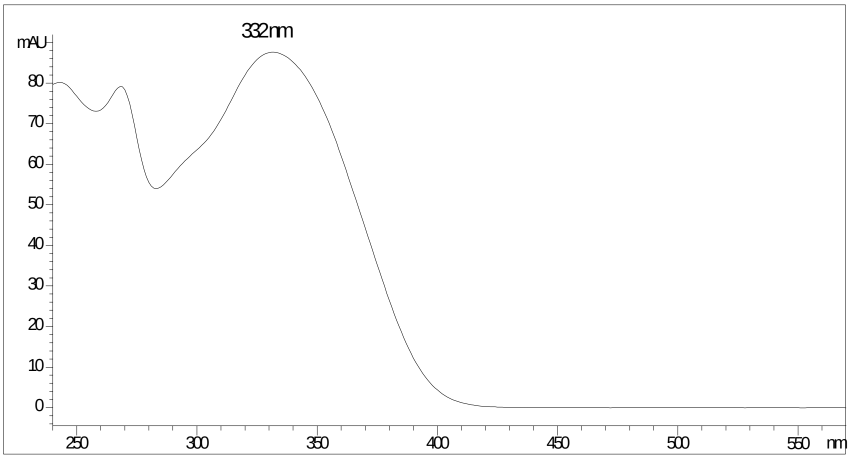

(b)

Figure S7: Examples of HPLC and MS spectra for phenolic compounds in methanol extract. (a) MS spectra and (b) DAD spectra for peak N in methanol extract.

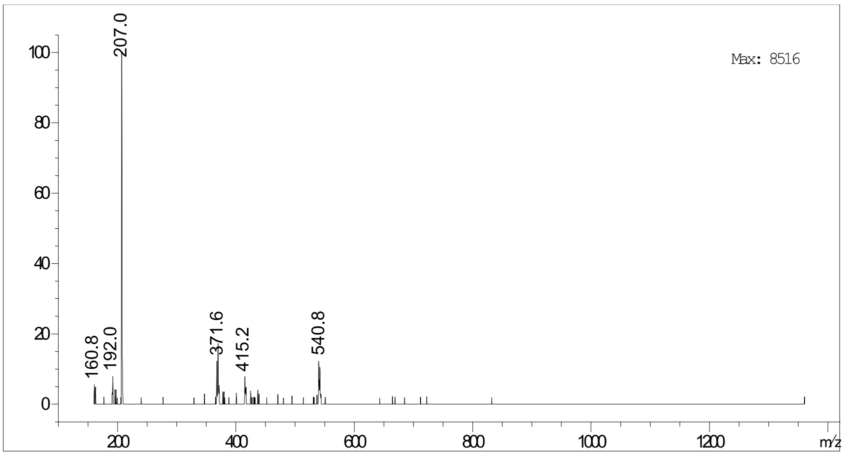

(a)

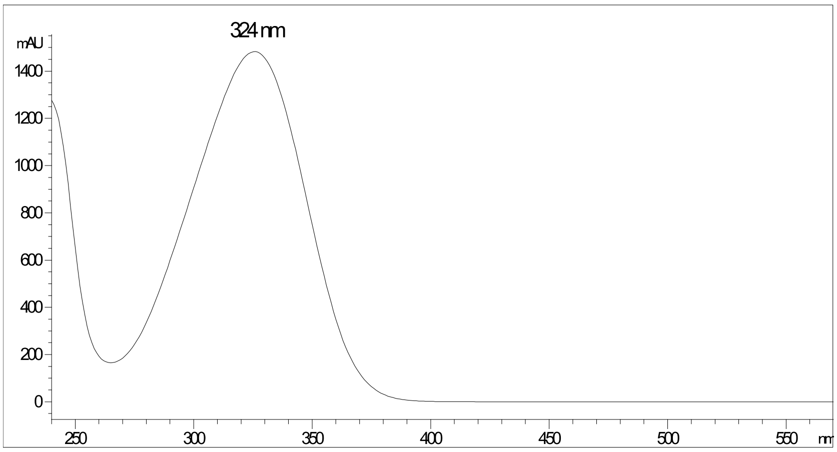

(b)

Figure S8: Examples of HPLC and MS spectra for phenolic compounds after alkaline hydrolysis. (a) MS spectra and (b) DAD spectra for peak 10 in alkaline hydrolysis.
